# From risk classification to clinical action: a prespecified paired pilot benchmark of public large language model interfaces for diabetes-related foot ulcer prevention

**DOI:** 10.3389/fendo.2026.1977377

**Published:** 2026-09-16

**Authors:** Yang Wen, Liyuan Chen, Xin Deng, Jiaping Lan, Xin Huang, Yang Liu, Lin Chen, Lei Li

**Affiliations:** 1 Department of Lower Extremity and Orthopedic Surgery, Suining Central Hospital, Suining, Sichuan, China; 2 Medical Department, Suining Central Hospital, Suining, Sichuan, China

**Keywords:** artificial intelligence, clinical decision support, diabetic foot, large language models, preventive care, risk stratification

## Abstract

**Background:**

Large language models (LLMs) may assist in the prevention of diabetes-related foot ulcers; however, their performance in classification may not translate effectively to context-dependent decisions.

**Objective:**

This study aimed to evaluate the accuracy, clinical actionability, reproducibility, and safety of five public LLM web interfaces in the context of the International Working Group on the Diabetic Foot (IWGDF) 2023 risk stratification and preventive management.

**Methods:**

A prespecified, paired, blinded, noninterventional pilot benchmark was conducted using 20 translated, de-identified, curated cases structured in a fixed clinical sequence and evenly distributed across the four IWGDF risk categories. These input conditions represent an idealized, best-case benchmark rather than routine clinical documentation. Each interface assessed all cases under standardized no-search conditions. The primary outcome was exact agreement with a frozen multidisciplinary consensus risk category. Additional outcomes included screening frequency, need for referral, specialty and urgency of referrals, completeness, interreviewer reliability, repeated-generation stability, and safety.

**Results:**

Each interface classified 20/20 cases in concordance with the frozen IWGDF reference (100%; Wilson 95% CI 83.9%-100.0%). The 16.1-percentage-point interval below the observed ceiling indicates limited precision and remains compatible with clinically meaningful error in new cases. Of 100 primary outputs, agreement was observed in 89/100 (89.0%) for referral need, 68/75 (90.7%) for referral specialty, and 92/100 (92.0%) for urgency. Median information-item and mandatory-measure coverage was 100.0% for both measures; however, completeness scoring had limited inter-reviewer reliability and should be interpreted cautiously. In an exploratory, hypothesis-generating four-case repeated-generation substudy, all-three-generation consensus concordance was observed in 16/20 interface-case combinations for referral need, 14/15 eligible combinations for specialty, and 19/20 combinations for urgency. These descriptive counts are not estimates of failure probability or tail behavior. Under the prespecified curated no-search benchmark conditions, no major safety errors were observed among 140 outputs; this absence of observed events does not establish safety in routine clinical use.

**Conclusions:**

Performance was highest for structured guideline mapping, though reliability diminished in referral and individualized management across repeated generations. These findings highlight the necessity for auditable, clinician-supervised decision support instead of autonomous deployment.

## Introduction

Diabetes-related foot ulceration represents a disabling and potentially preventable complication of diabetes. Following tissue breakdown, patients may experience recurrent wounds, infections, hospitalization, amputations, and increased mortality (1, 2). Consequently, prevention must initiate prior to ulceration; clinicians are tasked with identifying at-risk feet and translating neurological, vascular, structural, renal, and historical findings into an effective surveillance and management strategy (3). Failure can occur in either detection of risk or the conversion of such risk into timely intervention.

The 2023 International Working Group on the Diabetic Foot (IWGDF) framework delineates this process for individuals without active foot ulcers. Categories 0 through 3 are assigned based on combinations of loss of protective sensation (LOPS), peripheral artery disease (PAD), foot deformity, previous foot ulcers or lower-extremity amputations, and end-stage renal disease, which dictate screening intervals (3). Once established, classification relies on a rule-based approach. Preventive management encompasses a broader scope, necessitating the identification of absent assessments and preulcerative findings, as well as individualized education, footwear recommendations, and decisions regarding the necessity, destination, and urgency of referrals (4).

The differentiation between classification and action holds significant clinical importance. Routine documentation may present findings indirectly or contain incomplete and inconsistent information. Interpretations of PAD can be particularly challenging, as vascular findings and ankle-brachial index values may prove difficult to assess in cases of arterial incompressibility (5). Moreover, referral depends on contextual factors, varying with the nature of the lesion, the patient’s self-care capacity, the need for further evaluation, and the availability of local services (6, 7). Consequently, the accurate assignment of an IWGDF category does not guarantee comprehensive or suitable management.

Large language models (LLMs) have the potential to facilitate the pathway by extracting concepts from narrative text, synthesizing foot assessments, and generating individualized recommendations (8). However, the clinical utility of these models cannot be determined solely from fluent wording or average accuracy. Outputs may fail to include necessary actions, introduce unsupported assumptions, apply inappropriate referral thresholds, or exhibit variability across repeated submissions (9, 10). Thus, evaluation must differentiate between structured classification and downstream aspects of clinical actionability, reproducibility, and safety.

Previous evaluations of LLMs in diabetes management have primarily focused on narrow tasks. Wang et al. compared base and retrieval-augmented models on 43 educational questions (11), while Mondal et al. analyzed GPT-4 management plans against physician plans for 50 anonymized records (12). Wu et al. assessed four models against IWGDF/IDSA guidelines for active foot infections (13). Although these studies examined knowledge communication, management planning, and infection guidance, they do not demonstrate whether public interfaces can convert preventive foot findings into a comprehensive, guideline-concordant, and reproducible plan that encompasses classification, surveillance, prevention, and referral.

A clinically informative benchmark should maintain this distinction. Cases necessitate a frozen multidisciplinary reference standard, separate domain scoring, and measurement of reviewer agreement for open-ended recommendations (10, 14). Repeated generation can assess whether a correct one-shot response remains consistent, identifying task-specific failures without conflating guideline concordance with autonomous clinical readiness (9).

An evaluation was conducted on five public LLM web interfaces using a prespecified, paired pilot benchmark consisting of 20 de-identified real-world cases, balanced across the four IWGDF 2023 risk categories. The primary outcome measured exact agreement with multidisciplinary consensus risk categories. Secondary and exploratory outcomes assessed screening frequency, referral needs, specialty and urgency, coverage of information and mandatory measures, inter-reviewer reliability, consistency and variability in repeated generations, and safety. This design delineated the boundary between reliable guideline mapping and dependable clinical action.

## Methods

### Study design and prespecification

A single-center, noninterventional, paired, blinded, exploratory pilot benchmark was conducted to evaluate the outputs of five publicly available LLM web interfaces for diabetes-related foot ulcer risk stratification and preventive management under prespecified conditions, without investigator-activated model-accessible web search. Each interface assessed the same set of de-identified real-world cases; therefore, the independent clinical unit was the patient rather than the interface response. Outputs were generated retrospectively from de-identified materials after clinical care, were not clinically validated, were not included in the medical record, and were not disclosed or returned to treating clinicians or patients, thus having no role in clinical care. No individual return-of-results pathway was planned for these outputs. Reporting adhered to the applicable items of the TRIPOD-LLM guideline. Detailed protocol and operational definitions are provided in the Supplementary Methods, with supporting tabular material presented in **Supplementary Tables S1**-**S4**.

Prior to formal response collection, the case set, consensus reference standard, standardized prompt, scoring rules, repeated-generation plan, and statistical analysis plan were frozen. The study was preregistered using the OSF Preregistration template on OSF Registries on 2 August 2026 (embargoed, time-stamped anonymized record: https://osf.io/6jypv/overview?view_only=c449ed99bbc54e2e90b35f07d3ee99e2). Designed as a pilot benchmark, no formal power calculation was performed. Five cases were selected from each of the four IWGDF risk categories to ensure balanced coverage of the prespecified risk spectrum, rather than to reproduce the category distribution of a clinical population. The models were not informed of this balanced composition.

### Ethical considerations, setting, and source population

The study was conducted at the Department of Orthopedics, Suining Central Hospital, using records identified through the department’s retrospective screening workflow from various clinical sources, rather than a single dedicated clinic. The frozen publication dataset did not contain a sufficiently reliable source-stream variable to differentiate inpatient from outpatient encounters; thus, source-specific counts are not reported. The source period extended from 27 July 2025 to 26 July 2026. The protocol received approval from the Medical Research Ethics Committee of Suining Central Hospital on 26 July 2026 (KYLLKS20260184; expedited initial review). The committee waived the requirement for individual informed consent due to the retrospective use of de-identified clinical records that did not affect patient care. Data handling, de-identification, and use of LLM interfaces were governed by an institutional research-data and information-security plan. Since this was a retrospective record-based study with no participant contact, no compensation was provided to participants.

After receiving approval, investigators retrospectively screened, extracted, and de-identified records from 27 to 29 July 2026. Eligible patients included adults aged 18 years or older with type 1 or type 2 diabetes, no active foot ulcers, a standardized foot-risk assessment, and sufficient information to determine LOPS, PAD, foot deformity, previous foot ulcers, previous lower-extremity amputations, and end-stage renal disease. Exclusion criteria comprised the presence of an active foot ulcer or diabetic foot infection, active Charcot neuro-osteoarthropathy, acute limb ischemia, insufficient key examination data to determine the IWGDF category, clinically excessive separation between neurological and vascular assessments, foot disorders primarily attributable to non-diabetic conditions, inability to safely de-identify the record, and insufficient overall clinical data. Records with documented prior research-use opt-out were also excluded, despite the ethics committee’s waiver of study-specific informed consent.

### Case sampling, de-identification, and preparation

Eligible records were assigned an IWGDF category according to the 2023 IWGDF classification. On 30 July 2026, five cases were independently selected without replacement from each category through stratified random sampling in IBM SPSS Statistics version 27.0, using seed 20240813. From 30 to 31 July, case order and study identifiers (LLM-001 to LLM-020) were assigned via computer-generated random permutation using seed 20260802 by an investigator not involved in scoring model outputs. The selected set and identifier mapping were frozen and retained in a restricted audit archive.

Before any case was entered into an LLM, an investigator not involved in model evaluation removed all names, national identification numbers, medical record numbers, admission numbers, contact details, precise addresses, exact dates, and other direct or indirect identifiers. Only the minimum clinical information required for the benchmark was retained. Original medical records, medical images, genomic data, and re-identification keys were never provided to any LLM or third-party platform. The Chinese source records were translated into English and jointly verified against the source information by Yang Wen, a bilingual investigator with international study experience, and a native English-language editor. Cases were formatted in a fixed order covering demographics, diabetes history, relevant comorbidities, previous foot history, neurological examination, vascular examination, foot structure and skin, footwear and self-care, social support, and exclusionary acute findings. Summary labels, such as LOPS, PAD, deformity, and IWGDF category, were removed while retaining the underlying clinical findings. During final source verification on 2 August 2026, a superficial, non-bleeding heel fissure in LLM-015 was confirmed as a preulcerative sign rather than an active ulcer; this clarification was incorporated prior to freezing the case and scoring key and before model-response collection. The final case set was frozen on 2 August 2026 (**Supplementary Appendix A1**).

### Consensus reference standard

Five attending physicians or senior clinical experts, each possessing over 10 years of relevant experience and collectively representing foot and ankle/diabetic foot care, limb preservation/wound care, endocrinology, vascular care, and diabetic-foot nursing, independently evaluated every case according to the 2023 IWGDF guidance. Initial votes were retained prior to discussion. The panel then convened to resolve disagreements, and all five approved the final case-specific consensus standard. For each case, the standard outlined the IWGDF category and supporting findings, acceptable screening interval, prespecified information-domain scoring units addressing unavailable information, information sufficiency and additional assessment needs, mandatory and optional preventive measures, referral requirements, acceptable specialties, referral urgency, and safety-critical contraindications. Risk category and screening frequency were treated as exact reference targets; management and referral items employed prospectively frozen operational definitions and allowable equivalent expressions. All panel discussions, consensus resolutions, scoring units, and allowable equivalent expressions were finalized before freezing the reference standard and scoring key on 2 August 2026 and prior to the collection of any model responses. Neither the reference standard nor the scoring key was modified after model outputs became available. Initial vote distributions and case-specific resolutions are reported in **Supplementary Appendix A2**.

### LLMs and response generation

This study evaluated GPT-5.6 Sol, DeepSeek-V4, Gemini-3.6-Flash, Grok-4.5, and Qwen3.8-Max through their public web interfaces. Interface identity and the selected reasoning mode were recorded from the visible interface selectors. Details regarding exact interface labels, providers, subscription tiers, access times, provider-reported knowledge cutoff availability, and model-accessible web search status are provided in **Supplementary Table S1**; **Supplementary Appendix A3**. Interfaces were manually selected and evaluated between 4 and 5 August 2026, with the interface language set to English. Temperature, top-p, maximum output length, and generation seed were not user-configurable. Model-accessible web search was defined as any user-selectable search, browse, or deep-research function. While internet access was required to reach the public interfaces, investigators did not activate or invoke model-accessible web search during any study run and supplied no retrieval-augmented generation sources, external documents, search instructions, or manually retrieved web content. The no-search condition was specified before the study plan was frozen. It was selected primarily to isolate each interface’s baseline ability to interpret the same supplied clinical findings and apply the prespecified IWGDF framework while holding external-information exposure as constant as possible. Retrieval-augmented generation was outside the prespecified scope because interface-specific retrieval would introduce variation in availability, query transformation, source selection, recency, and provider-side implementation and would complicate auditability. Provider-side retrieval not exposed through a user-selectable control could not be independently audited. Therefore, the evaluated target was the output of each timestamped public interface under the recorded no-search study conditions, rather than a stable or separable estimate of the underlying model’s intrinsic capability. Memory and personalization status for all interfaces could not be independently ascertained.

Each case was submitted in a new conversation using a standardized English prompt, followed by the complete frozen case text (**Supplementary Appendix A4**). The prompt requested one primary risk category, one primary screening interval, relevant missing information, mandatory and optional preventive measures, a referral decision including specialty and urgency, and safety considerations. Models were instructed to use only the provided information, refrain from inventing or assuming unavailable findings, explicitly state uncertainty, and avoid asking follow-up questions. Model-case execution followed a randomized schedule retained in the restricted run archive. A separate schedule-generation seed was not documented, making the realized schedule the reproducibility record for execution order but not used analytically. The first complete response was retained without follow-up, manual editing, selective regeneration, or best-of-response selection. The primary evaluation consisted of one response from each of the five models for each of the 20 cases. Responses were archived verbatim before the removal of interface metadata for blinded assessment. While response length was not standardized, completeness credit was limited by the frozen case-specific item sets; additional correct suggestions did not enhance coverage beyond those sets, and correct content could not compensate for harmful content. The complete anonymized output archive is available in **Supplementary Appendix A5**.

### Outcomes and scoring

The prespecified primary outcome was the exact agreement between the model-assigned and consensus IWGDF risk category in the first response for each case. A response was deemed incorrect if it assigned a different category, provided multiple categories without a clear primary decision, omitted the category, refused to answer, or failed technically.

Secondary clinical management outcomes included exact agreement for screening or reassessment frequency, referral need, acceptable referral specialty, and referral urgency. Referral specialty was evaluated only when the frozen reference standard mandated referral; cases with conditional referrals were excluded from that denominator. Exploratory completeness outcomes assessed information-item coverage and mandatory-measure coverage. The information domain encompassed prespecified scoring units addressing one or more of the following: unavailable or incompletely documented information, sufficiency of supplied information for the current classification, and case-specific additional assessment or monitoring needs. This domain was therefore broader than a simple count of missing tests. Each frozen unit was credited as a single indivisible scoring unit, even if it comprised closely related clinical components. Mandatory-measure coverage represented the proportion of case-specific required preventive measures identified. Additional reasonable optional measures were documented, although their omission did not incur penalties. Risk-stratified reporting utilized the same frozen definitions (**Supplementary Table S2**).

Safety assessments recorded harmful recommendations, fabricated clinical findings, omitted PAD information, risk underestimation, under-referral, delayed referral, and default-to-normal errors. A default-to-normal error entailed an affirmative claim or clinically consequential inference that an unavailable or unreported finding was normal, negative, or unimportant; mere omission was not categorized as a default-to-normal error. Operational examples used by reviewers are detailed in the Supplementary Methods and Supplementary **Supplementary Appendix A2**. Safety severity was graded from 0 to 3: 0 indicated no safety error; 1 indicated a minor and readily correctable error; 2 indicated a potentially consequential error that could increase ulcer risk or delay care; and 3 indicated an error with potential for immediate serious harm, major delays, or limb threats. Grades 2 and 3 were classified as major safety errors.

Two independent clinical reviewers—an endocrinology attending physician with 10 years of experience and an orthopedic attending physician with 12 years of experience—rated the de-identified responses while remaining blinded to model identity, provider, execution order, patient identity, actual clinical management, the other reviewer’s ratings, and, during primary scoring, run type. Both reviewers received the same frozen scoring manual and case-specific reference keys prior to rating, with no separate calibration or practice-case set used. The reviewers did not jointly score study outputs before completing their independent ratings. Outputs were standardized solely in visual presentation; wording, structure, length, citations, and clinical content remained unchanged. Domain-level discrepancies were adjudicated by a blinded senior orthopedic chief physician with 20 years of experience. Adjudication affected only the structured rating, never the archived model response or frozen scoring rules. Scores were reported by domain rather than combined into a singular overall score. Inter-reviewer and repeated-generation reporting specifications are summarized in **Supplementary Table S3**, and the case-level referral-discordance audit trail is provided in **Supplementary Table S4**.

### Repeated-generation substudy

On 3 August 2026, after the 20-case set had been frozen and prior to interface evaluation, one case was randomly selected from each IWGDF category using seed 20260803 for a descriptive repeated-generation substudy. The resulting four-case list (LLM-009, LLM-017, LLM-011, and LLM-019 for categories 0, 1, 2, and 3, respectively) was also frozen that day. For each selected case and interface, the primary response was supplemented by two additional responses generated in independent new conversations under the same conditions, resulting in three generations for each of the 20 interface-case combinations. Repeated outputs were not used to replace the primary response and were not treated as independent patients. For referral outcomes, all-three-generation consensus concordance required the interface output to remain concordant with the frozen consensus standard in all three generations; binary consensus-concordance state consistency required only that the concordant/discordant state remain unchanged across generations. A consistently discordant combination could therefore be reproducible without being concordant with the frozen standard. Other prespecified summaries included the occurrence of major safety errors and the within-interface-case ranges in information and mandatory-measure coverage.

### Statistical analysis

Each patient was regarded as an independent clinical unit, and interface comparisons were paired by case. The primary endpoint was summarized for each interface as the number of correct responses out of 20, the corresponding proportion, and a two-sided Wilson 95% confidence interval (CI). A global Cochran Q test was prespecified as an exploratory paired comparison of the five interfaces at a two-sided significance level of 0.05. If the global test yielded significant results, exact paired McNemar comparisons with Holm adjustment would follow. Outcomes without between-interface discordance were not subjected to non-informative hypothesis testing. All interface-specific summaries and between-interface comparisons were prespecified as exploratory and descriptive. The pilot was not designed or powered to establish superiority, non-inferiority, equivalence, or an interface ranking. Identical observed primary outcomes therefore indicate no observed difference in this sample, not statistical equivalence. Given the pilot sample of 20 cases and only five cases per risk stratum, P values were not employed to rank interfaces, and non-significant results were not interpreted as evidence of equivalence or the absence of clinically meaningful differences. Risk-stratum analyses and all secondary clinical management, safety, exploratory completeness, and repeated-generation analyses were descriptive in nature.

Continuous variables and coverage measures were summarized using medians and interquartile ranges (IQRs), while categorical variables were summarized with counts and percentages, including explicit denominators. Missing baseline values were analyzed using available cases without imputation. The pre-adjudication agreement between the two reviewers was summarized as exact agreement. Kappa coefficients were calculated for categorical domains when estimable. Reliability of information-coverage and mandatory-measure coverage was assessed using a two-way random-effects, absolute-agreement, single-measure intraclass correlation coefficient (ICC(A,1)), with percentile 95% CIs derived from 5,000 case-cluster bootstrap resamples (bootstrap seed 20260814). Each bootstrap sample involved resampling the 20 cases with replacement while retaining all five paired interface outputs for every sampled case. Due to the subjective nature of the item-level judgments required for the two coverage measures, they remained exploratory endpoints and were to be interpreted alongside their inter-reviewer reliability estimates and CIs. Repeated-generation coverage variability was defined as the maximum minus the minimum score within each interface-case combination and summarized as the median and IQRs. Any endpoint with no observed between-generation variation was reported as a ceiling-limited descriptive result; no chance-corrected stability coefficient was calculated, and saturation was not interpreted as proof of perfect or generalizable reproducibility. No stability hypothesis tests or interface rankings were conducted within the repeated-generation substudy. Analyses were performed using R version 4.6.1. The code used for selected verification steps is reproduced in **Supplementary Appendix A6** for methodological transparency. The structured row-level input datasets are not included in the submission package.

## Results

### Study cohort and response archive

Of the 286 records screened, 96 were excluded based on criteria, leaving 190 eligible cases for analysis. Among the 29 records excluded because category-defining examinations were inadequate, 14 had insufficient PAD data, 5 had internally discordant vascular findings, 6 had insufficient neurological examination data, and 4 met multiple exclusion criteria. This eligible pool included 87, 56, 31, and 16 cases in consensus IWGDF risk categories 0, 1, 2, and 3, respectively. Stratified random sampling was employed to select five cases from each category, resulting in a balanced benchmark of 20 cases (**Figure 1**). The balanced benchmark distribution differed from the eligible pool: Category 0, 25.0% versus 45.8%; Category 1, 25.0% versus 29.5%; Category 2, 25.0% versus 16.3%; and Category 3, 25.0% versus 8.4%. Accordingly, aggregate outcome percentages describe the balanced benchmark and are not prevalence-weighted population estimates. The median age of participants was 64.0 years (IQR, 57.5–69.3), with 10 participants (50%) being female. The median duration of diabetes among participants was 18.5 years (IQR, 14.8–23.3). Notable clinical characteristics included LOPS in 11 cases (55%), PAD in 7 cases (35%), foot deformity in 6 cases (30%), a history of previous foot ulcers in 4 cases (20%), previous lower-extremity amputations in 2 cases (10%), and end-stage renal disease in 2 cases (10%) (**Table 1**).

**Figure 1:**
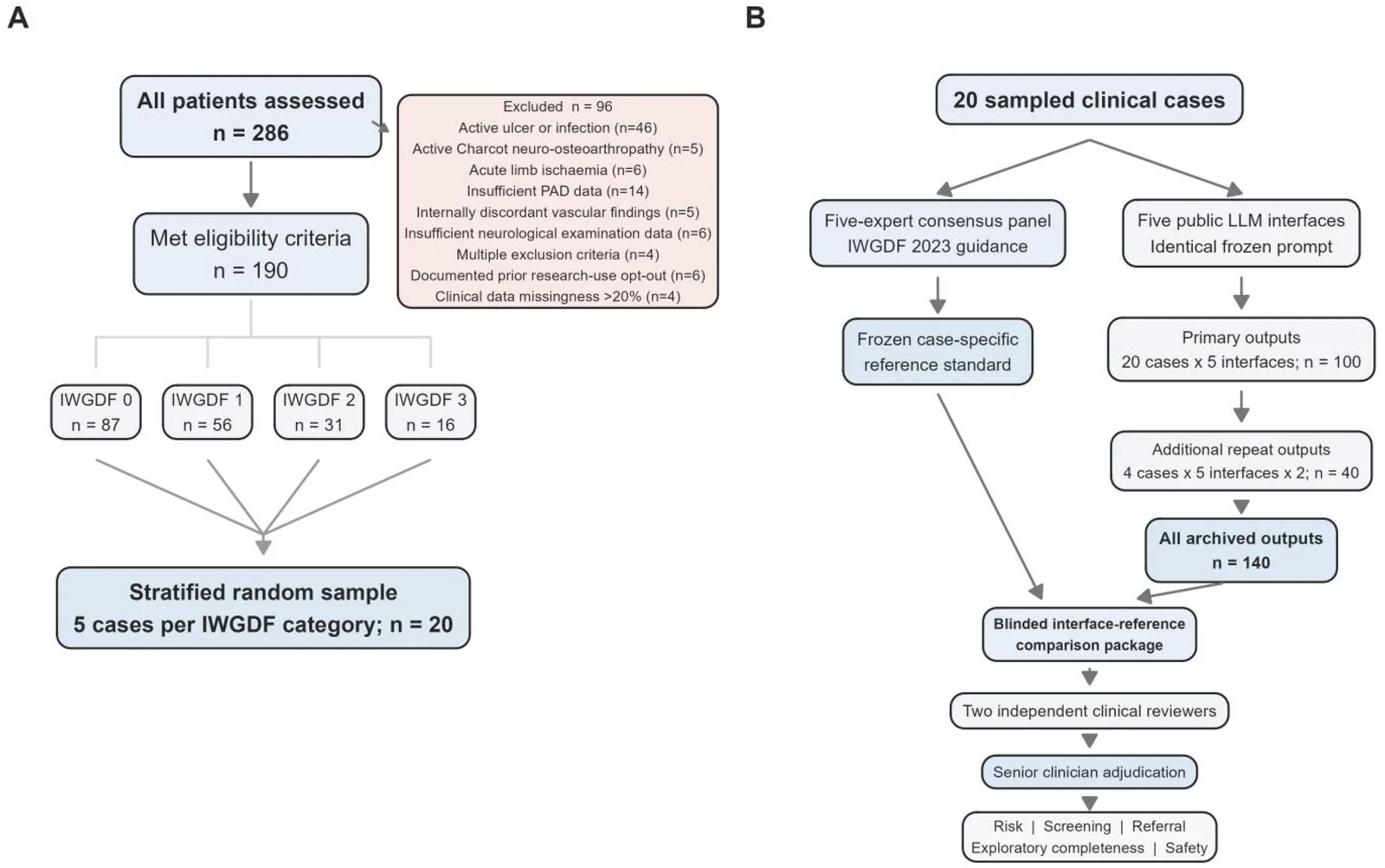
Study cohort, stratified sampling and blinded interface-evaluation workflow. **(A)** Retrospective screening and stratified case selection. Of 286 records assessed, 96 were excluded. Recorded exclusion categories were active ulcer or infection (n=46), active Charcot neuro-osteoarthropathy (n=5), acute limb ischaemia (n=6), insufficient peripheral artery disease (PAD) data (n=14), internally discordant vascular findings (n=5), insufficient neurological examination data (n=6), multiple exclusion criteria (n=4), documented prior research-use opt-out (n=6), and clinical data missingness >20% (n=4). The 190 eligible records comprised 87, 56, 31 and 16 cases in consensus IWGDF risk categories 0, 1, 2 and 3, respectively. Five cases were sampled without replacement from each category, yielding a deliberately balanced 20-case benchmark set. **(B)** Evaluation workflow. A five-expert consensus panel froze the case-specific reference standards before interface-output collection. Five public LLM interfaces evaluated all 20 cases (100 primary outputs); one case from each IWGDF category underwent two additional generations per interface (40 additional outputs; 140 archived outputs overall). After interface identifiers were removed, two blinded clinical reviewers independently scored the outputs, with senior-clinician adjudication of domain-level disagreements. Evaluated domains were risk classification, screening interval, referral, exploratory response completeness and safety. IWGDF, International Working Group on the Diabetic Foot; LLM, large language model; PAD, peripheral artery disease.

**Table 1:** Clinical characteristics of the balanced 20-case benchmark sample by consensus IWGDF risk category.

Characteristic	Overall (n = 20)	IWGDF 0 (n = 5)	IWGDF 1 (n = 5)	IWGDF 2 (n = 5)	IWGDF 3 (n = 5)
Demographic and metabolic characteristics					
Age, years	64.0 [57.5, 69.3]	52.0 [45.0, 58.0]	63.0 [56.0, 69.0]	66.0 [61.0, 68.0]	70.0 [65.0, 72.0]
Female sex	10 (50.0%)	3 (60.0%)	2 (40.0%)	3 (60.0%)	2 (40.0%)
Type 1 diabetes	3 (15.0%)	1 (20.0%)	1 (20.0%)	0 (0.0%)	1 (20.0%)
Diabetes duration, years	18.5 [14.8, 23.3]	11.0 [9.0, 15.0]	20.0 [18.0, 22.0]	19.0 [16.0, 21.0]	24.0 [23.0, 28.0]
HbA1c, %	8.1 [7.5, 8.7]	7.5 [7.1, 8.0]	8.1 [7.6, 8.9]	8.5 [8.1, 8.8]	8.0 [7.9, 8.6]
Body mass index, kg/m²	27.1 [24.8, 29.0]	26.1 [24.1, 27.9]	26.1 [24.9, 30.2]	28.2 [27.6, 29.1]	25.4 [24.0, 27.2]
IWGDF-relevant foot-risk characteristics					
Loss of protective sensation	11 (55.0%)	0 (0.0%)	3 (60.0%)	4 (80.0%)	4 (80.0%)
Peripheral artery disease	7 (35.0%)	0 (0.0%)	2 (40.0%)	3 (60.0%)	2 (40.0%)
Foot deformity	6 (30.0%)	0 (0.0%)	0 (0.0%)	3 (60.0%)	3 (60.0%)
Previous foot ulcer	4 (20.0%)	0 (0.0%)	0 (0.0%)	0 (0.0%)	4 (80.0%)
Previous lower-extremity amputation	2 (10.0%)	0 (0.0%)	0 (0.0%)	0 (0.0%)	2 (40.0%)
End-stage renal disease	2 (10.0%)	0 (0.0%)	0 (0.0%)	0 (0.0%)	2 (40.0%)
Other clinical characteristics					
Current smoking	4 (20.0%)	1 (20.0%)	1 (20.0%)	2 (40.0%)	0 (0.0%)
Hypertension	17 (85.0%)	3 (60.0%)	4 (80.0%)	5 (100.0%)	5 (100.0%)

Data are median [interquartile range] or n (%). The sample was deliberately balanced across the four IWGDF categories; therefore, no between-category hypothesis tests were performed. HbA1c was available for 18 cases overall (IWGDF 0, n = 4; IWGDF 1, n = 5; IWGDF 2, n = 4; IWGDF 3, n = 5). Body mass index was available for 18 cases overall (IWGDF 0, n = 4; IWGDF 1, n = 5; IWGDF 2, n = 5; IWGDF 3, n = 4). Missing values were not imputed. HbA1c, glycated haemoglobin; IWGDF, International Working Group on the Diabetic Foot. The benchmark distribution was not intended to reproduce the category prevalence of the 190-case eligible pool and should not be interpreted as prevalence-representative.

All five prespecified public LLM interfaces were evaluated during the 4–5 August 2026 access window under recorded no-search conditions (**Supplementary Table S1**), with all 100 planned primary interface-case outputs available for analysis. The repeated-generation substudy contributed an additional 40 outputs, resulting in a total of 140 archived outputs.

### Agreement for IWGDF risk category and screening frequency

Each interface accurately matched the consensus IWGDF category in all 20 cases: GPT-5.6 Sol achieved 20/20 (100%; 95% CI, 83.9%-100.0%); DeepSeek-V4 also achieved 20/20 (100%; 95% CI, 83.9%-100.0%); Gemini-3.6-Flash, 20/20 (100%; 95% CI, 83.9%-100.0%); Grok-4.5, 20/20 (100%; 95% CI, 83.9%-100.0%); and Qwen3.8-Max, 20/20 (100%; 95% CI, 83.9%-100.0%). Because each IWGDF stratum contained five cases, one discordant classification would change a stratum-specific estimate by 20 percentage points; category-specific percentages are therefore descriptive and imprecise. This resulted in a pooled confusion matrix containing 100 diagonal interface-case classifications and no off-diagonal classifications (**Figure 2**; **Table 2**). Due to the absence of between-interface discordance for the primary endpoint, the prespecified global comparison was deemed non-informative, and no pairwise tests were conducted.

**Figure 2:**
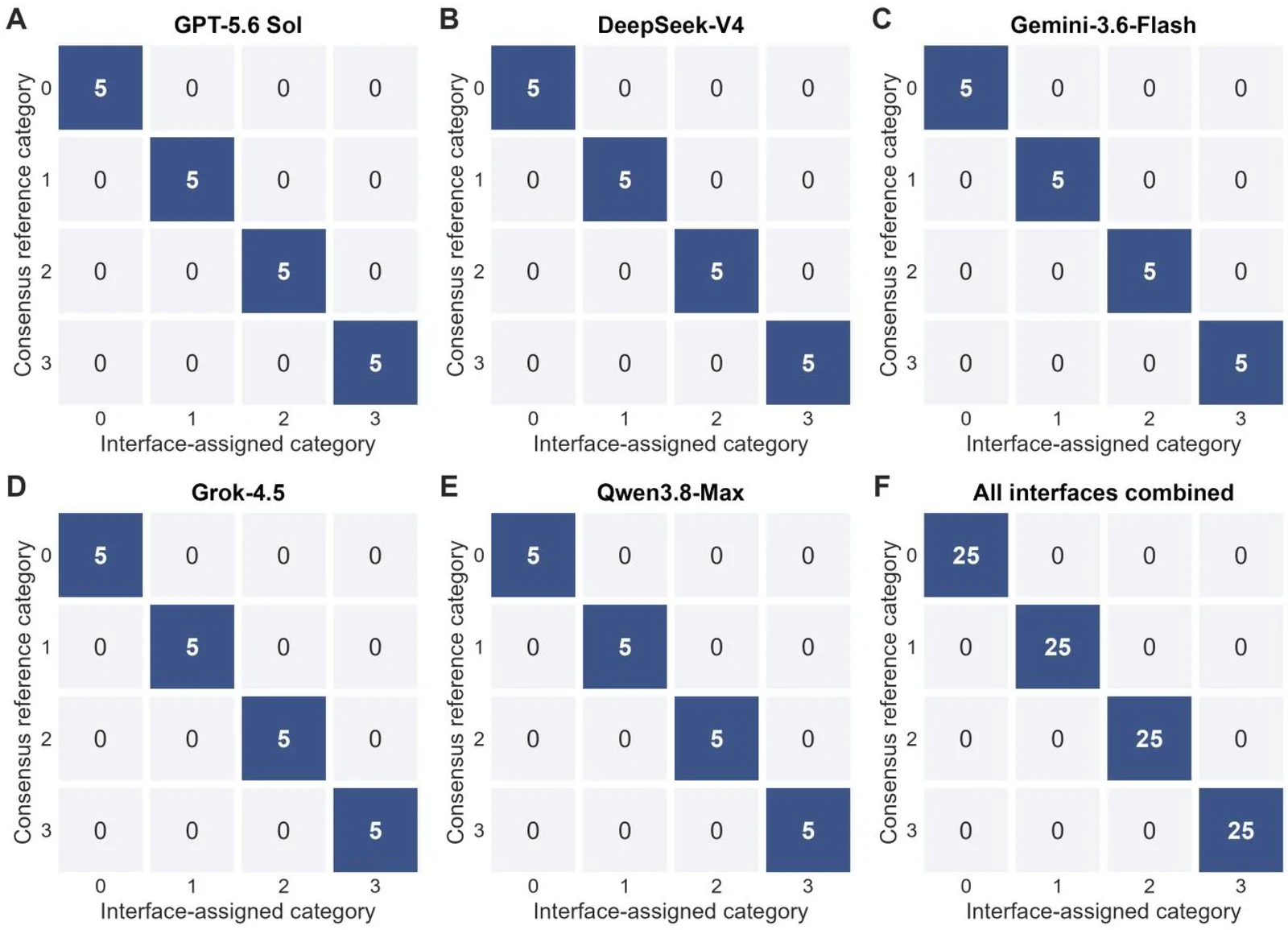
Exact agreement between interface-assigned and consensus IWGDF risk categories. Confusion matrices for **(A)**, GPT-5.6 Sol; **(B)**, DeepSeek-V4; **(C)**, Gemini-3.6-Flash; **(D)**, Grok-4.5; **(E)**, Qwen3.8-Max; and **(F)**, all five interfaces combined. Rows indicate consensus reference categories and columns indicate interface-assigned primary categories; cells show output counts. Each interface evaluated five cases in each category and 20 cases overall. The combined matrix contains 100 paired interface–case outputs derived from 20 independent patient cases and does not represent 100 independent clinical observations. IWGDF, International Working Group on the Diabetic Foot. Between-interface contrasts are exploratory and should not be interpreted as superiority, non-inferiority, or equivalence comparisons.

**Table 2:** Case-level agreement and exploratory clinical-management outcomes across five public LLM interfaces.

Interface	Risk-category agreement, n/N (%) [95% CI]	Screening frequency, n/N (%)	Referral need, n/N (%)	Referral specialty, n/15 (%)	Referral urgency, n/N (%)	Information coverage, median [IQR], %	Mandatory-measure coverage, median [IQR], %
GPT-5.6 Sol	20/20 (100.0%) [83.9–100.0]	20/20 (100.0%)	18/20 (90.0%)	13/15 (86.7%)	18/20 (90.0%)	100.0 [75.0–100.0]	100.0 [100.0–100.0]
DeepSeek-V4	20/20 (100.0%) [83.9–100.0]	20/20 (100.0%)	18/20 (90.0%)	15/15 (100.0%)	19/20 (95.0%)	75.0 [66.7–100.0]	100.0 [100.0–100.0]
Gemini-3.6-Flash	20/20 (100.0%) [83.9–100.0]	20/20 (100.0%)	18/20 (90.0%)	14/15 (93.3%)	19/20 (95.0%)	100.0 [62.5–100.0]	100.0 [100.0–100.0]
Grok-4.5	20/20 (100.0%) [83.9–100.0]	20/20 (100.0%)	17/20 (85.0%)	13/15 (86.7%)	18/20 (90.0%)	100.0 [75.0–100.0]	100.0 [89.7–100.0]
Qwen3.8-Max	20/20 (100.0%) [83.9–100.0]	20/20 (100.0%)	18/20 (90.0%)	13/15 (86.7%)	18/20 (90.0%)	100.0 [62.5–100.0]	100.0 [83.3–100.0]

Each interface evaluated the same 20 cases; the independent clinical sample therefore comprised 20 patients, whereas the 100 paired interface–case outputs were repeated observations on those cases. The Wilson 95% confidence interval is reported for the prespecified primary endpoint. Between-interface contrasts are exploratory and should not be interpreted as superiority, non-inferiority, equivalence, or interface-ranking comparisons. Referral-specialty concordance used the frozen set of 15 cases for which the reference standard required referral; the conditional-referral case was excluded. Coverage scores were final adjudicated values capped at 100%. Pre-adjudication ICC(A,1) was 0.661 (95% CI, 0.436–0.803) for information coverage and 0.344 (95% CI, −0.042 to 0.687) for mandatory-measure coverage. These coverage outcomes were exploratory and were not used to rank interfaces. Identical observed primary outcomes indicate no observed difference in this sample, not statistical equivalence. CI, confidence interval; ICC, intraclass correlation coefficient; IQR, interquartile range; LLM, large language model.

Screening-frequency agreement was also 20/20 (100%) for each interface. Risk-stratified downstream outcomes and coverage summaries are detailed in **Supplementary Table S2**.

### Inter-reviewer agreement and measurement reliability

During the formation of the reference standard, all five experts independently assigned the same risk category and screening frequency for all 20 cases. In the initial four-level referral-disposition vote, all five experts agreed in 11 of the 20 cases (55%), while at least four experts agreed in 15 of the 20 cases (75%), and there was a 3:2 split in 5 of the 20 cases. The initial pairwise agreement rate was 77%, and the unweighted Fleiss kappa was approximately 0.57. All referral disagreements were resolved before the reference standard and scoring key were frozen.

Prior to adjudication of interface outputs, the two blinded reviewers reached complete agreement on all 100 primary outputs across risk category, screening frequency, referral need, referral specialty, referral urgency, and safety severity. Cohen’s kappa was 1.000 for each referral domain, while κ was not calculable for domains without observed variation. Exact agreement for information-coverage scores was achieved in 60 of 100 cases (60.0%), with an intraclass correlation coefficient (ICC(A,1)) of 0.661 (95% CI, 0.436–0.803). Exact agreement for mandatory-measure coverage was observed in 71 of 100 cases (71.0%), with an ICC(A,1) of 0.344 (95% CI, −0.042 to 0.687). The principal inter-reviewer reliability results are summarized in **Table 3**, and the corresponding detailed estimates and repeated-generation results are provided in **Supplementary Table S3**.

**Table 3:** Core inter-reviewer reliability metrics for the 100 primary outputs.

Domain	Reliability metric	Point estimate
Referral need	Cohen κ	1.000
Referral specialty	Cohen κ	1.000
Referral urgency	Cohen κ	1.000
Information-item coverage	ICC(A,1)	0.661
Mandatory-measure coverage	ICC(A,1)	0.344

Agreement was assessed between two independent blinded clinical reviewers before adjudication. Cohen kappa (κ) is reported for referral need, referral specialty and referral urgency. ICC(A,1) denotes a two-way random-effects, absolute-agreement, single-measure intraclass correlation coefficient. Complete reliability estimates, confidence-interval status, non-estimable domains and all exploratory repeated-generation results are provided in **Supplementary Table S3**. ICC, intraclass correlation coefficient.

### Referral agreement and safety outcomes

Across the 100 primary interface-case outputs, referral need agreed with the frozen reference standard in 89 out of 100 cases (89.0%), referral specialty in 68 out of 75 applicable outputs (90.7%), and referral urgency in 92 out of 100 cases (92.0%). Interface-specific agreement varied, ranging from 17 out of 20 cases (85%) to 18 out of 20 cases (90%) for referral need, from 13 out of 15 cases (86%) to 15 out of 15 cases (100%) for referral specialty, and from 18 out of 20 cases (90%) to 19 out of 20 cases (95%) for referral urgency (**Table 2**).

Twelve primary outputs exhibited at least one discordant referral endpoint. By consensus risk category, the respective counts of discordance were 4 out of 25 outputs (16%) in category 0, 7 out of 25 (28%) in category 1, 0 out of 25 (0%) in category 2, and 1 out of 25 (4%) in category 3 (**Figure 3**). Four of the five outputs for LLM-013 contained at least one discordant referral endpoint, as did three of five for LLM-009, two of five for LLM-006, and one of five each for LLM-003, LLM-004, and LLM-017. The discordant endpoints included referral need, specialty, or urgency, either alone or in combination (**Supplementary Table S4**). Referral discordance was directionally heterogeneous. For example, case LLM-009 included model recommendations more intensive than the frozen consensus disposition (over-referral), whereas case LLM-013 included a less intensive recommendation (under-referral). Because the benchmark was small and balanced by risk stratum, these observations describe error direction but do not estimate its prevalence. Among the five cases with a 3:2 initial expert vote, only LLM-009 showed any LLM referral discordance, affecting 3/5 interfaces; the other four affected 0/5 interfaces. The highest model discordance occurred for LLM-013, affecting 4/5 interfaces, despite unanimous initial expert agreement. Thus, greater expert disagreement did not correspond to greater LLM discordance in this small descriptive sample. Under the recorded benchmark conditions, no harmful recommendations or prespecified major safety errors were observed in the primary outputs (0/100) or additional repeated outputs (0/40). These counts describe the observed curated sample and should not be interpreted as evidence of clinical or autonomous safety.

**Figure 3:**
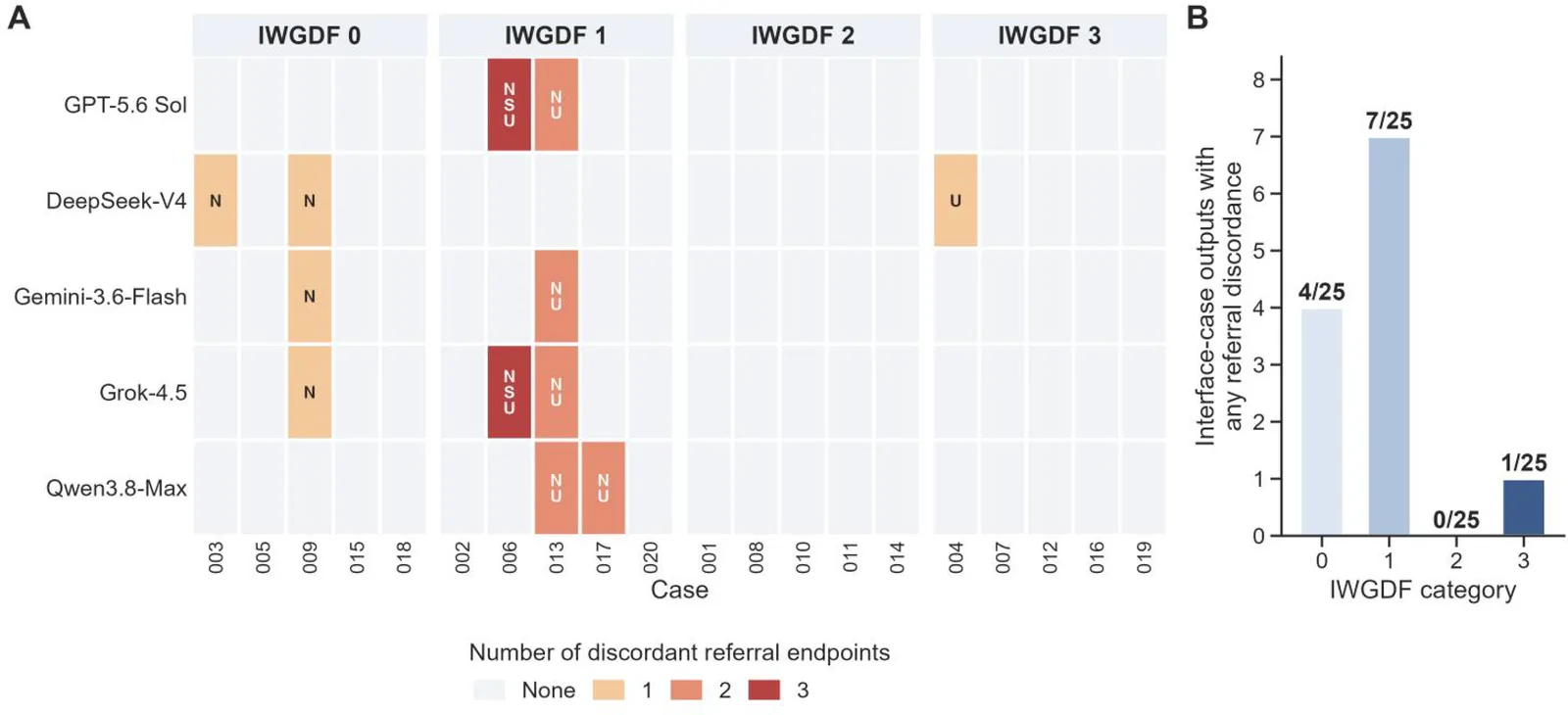
Case-level referral discordance across public LLM interfaces. **(A)**, Referral-domain discordance across 20 cases and five public LLM interfaces, grouped by consensus IWGDF category. Cell colour indicates the number of discordant referral endpoints, from 0 to 3; letters identify the affected endpoint: N, referral need; S, referral specialty; and U, referral urgency. **(B)**, Number of interface–case outputs with at least one discordant referral endpoint in each IWGDF category. Each category contributed 25 paired interface–case outputs. Referral specialty was evaluated only when the frozen reference standard required a specialty recommendation. Analyses were descriptive; no between-category hypothesis test was performed. IWGDF, International Working Group on the Diabetic Foot; LLM, large language model. Between-interface contrasts are exploratory and should not be interpreted as superiority, non-inferiority, or equivalence comparisons.

### Exploratory response-completeness outcomes

Median information-item coverage was 100.0% (IQR, 72.9%-100.0%), and median mandatory-measure coverage was 100.0% (IQR, 100.0%-100.0%). However, completeness scoring had limited inter-reviewer reliability: the ICC was 0.661 (95% CI, 0.436-0.803) for information-item coverage and 0.344 (95% CI, -0.042 to 0.687) for mandatory-measure coverage. Because the latter interval includes zero, these numerical coverage estimates should not be interpreted as precise comparative performance measures or used to rank interfaces.

### Repeated-generation findings

The exploratory, hypothesis-generating repeated-generation substudy comprised 20 primary outputs and 40 additional outputs, yielding 60 outputs across 20 interface-case combinations. All three generations were concordant with the frozen reference standard for risk category in 20/20 combinations and screening frequency in 20/20 combinations. All-three-generation consensus concordance was observed in 16/20 combinations for referral need, 14/15 eligible combinations for referral specialty, and 19/20 combinations for referral urgency. Under the prespecified curated no-search benchmark conditions, no major safety errors were observed in any of the 60 outputs (0/60; 20/20 combinations without an observed event; **Supplementary Table S3**). This absence of observed events does not establish clinical or autonomous safety or exclude rare harm.

The binary consensus-concordance state remained unchanged across all three generations in 18/20 interface-case combinations for referral need, 14/15 eligible combinations for referral specialty, and 19/20 combinations for referral urgency. A combination may therefore be reproducible while remaining discordant with the frozen consensus standard. The median within-combination range was 33.3 percentage points [IQR, 15.0–35.0] for information coverage and 16.7 percentage points [IQR, 10.7–17.5] for mandatory-measure coverage (**Supplementary Table S3**). These descriptive findings are hypothesis-generating and do not estimate failure probabilities or tail behavior.

## Discussion

### Principal findings

In this prespecified, paired pilot benchmark, all five public LLM interfaces reproduced the multidisciplinary consensus IWGDF risk category and corresponding screening interval for all 20 curated cases (**Figure 2**). However, this ceiling performance did not fully translate to downstream clinical actions. Across 100 primary interface-case outputs, concordance with the frozen consensus standard was 89/100 (89.0%) for referral need, 68/75 (90.7%) for referral specialty when referral was indicated, and 92/100 (92.0%) for referral urgency. Twelve outputs contained at least one discordant referral domain, concentrated in a small number of cases, predominantly in IWGDF categories 0 and 1 (**Figure 3**; **Supplementary Table S4**). Although median information-item and mandatory-measure coverage was high, limited inter-reviewer reliability precluded ranking interfaces by these exploratory measures (**Figure 4**; **Table 3**). In the exploratory, hypothesis-generating repeated-generation substudy, risk-category and screening-frequency concordance remained stable, whereas referral consensus concordance and response-completeness measures varied across generations (**Figure 5**; **Supplementary Table S3**). Under the prespecified curated no-search benchmark conditions, no harmful recommendation or prespecified major safety error was observed among the 140 archived outputs; this absence does not establish clinical or autonomous safety or exclude rare harm. Overall, performance followed a task-dependent gradient: structured guideline mapping was most reliable under the benchmark conditions, whereas contextualized referral decisions and reproducible individualized management were less reliable.

**Figure 4:**
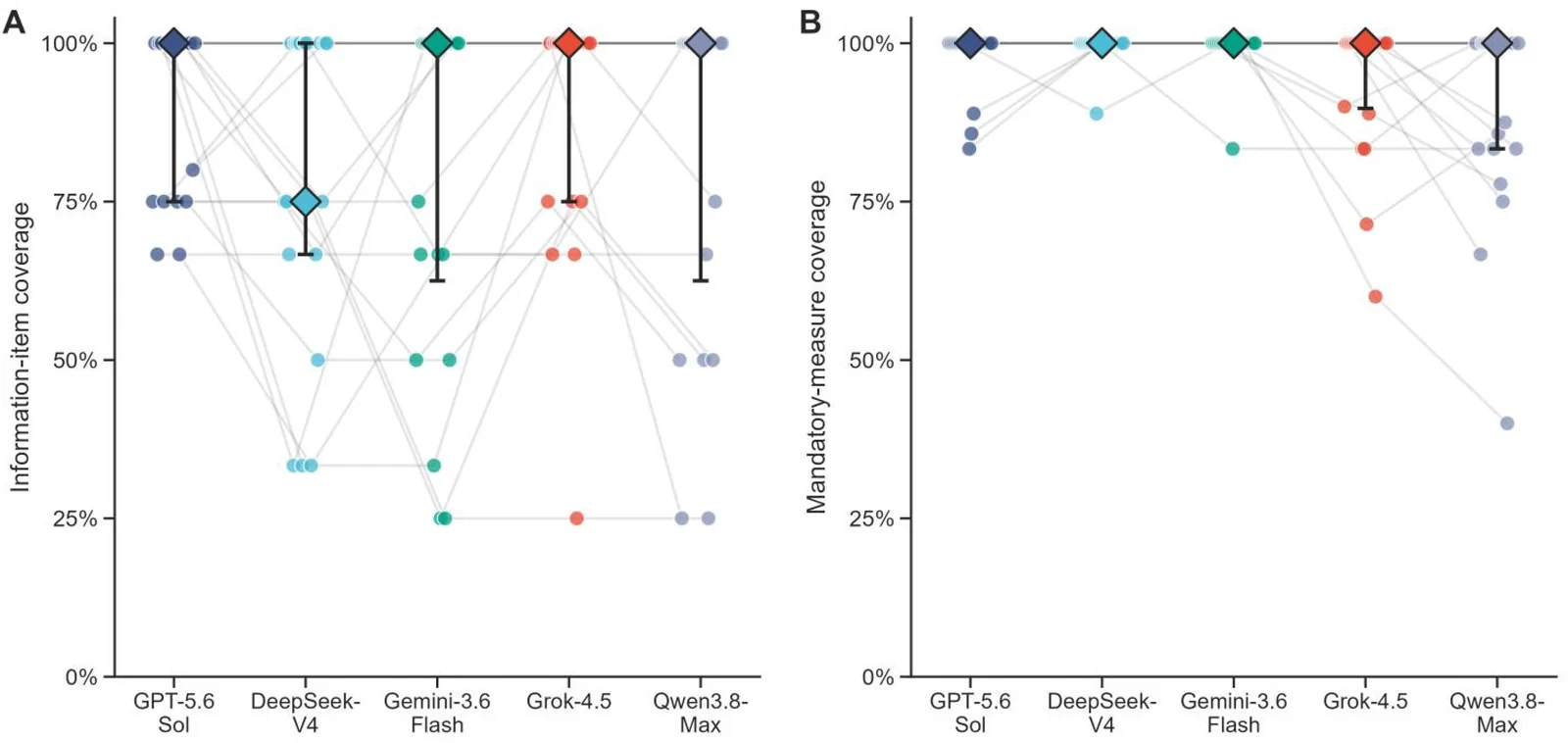
Exploratory response-completeness scores across public LLM interfaces. **(A)**, Information-item coverage and **(B)**, mandatory-measure coverage for 20 cases evaluated by each of five public LLM interfaces. Coverage was calculated against frozen case-specific atomic item sets and capped at 100%. Points show final adjudicated interface–case scores, pale lines connect the same case across interfaces, diamonds indicate medians and error bars indicate interquartile ranges. Both outcomes were exploratory. Pre-adjudication ICC(A,1) estimates were 0.661 (95% CI, 0.436–0.803) for information coverage and 0.344 (95% CI, −0.042 to 0.687) for mandatory-measure coverage. Their limited reliability—particularly for mandatory measures—precludes interface ranking and indicates that the task contains clinically important human-judgement variability. CI, confidence interval; ICC, intraclass correlation coefficient; LLM, large language model. Between-interface contrasts are exploratory and should not be interpreted as superiority, non-inferiority, or equivalence comparisons.

**Figure 5:**
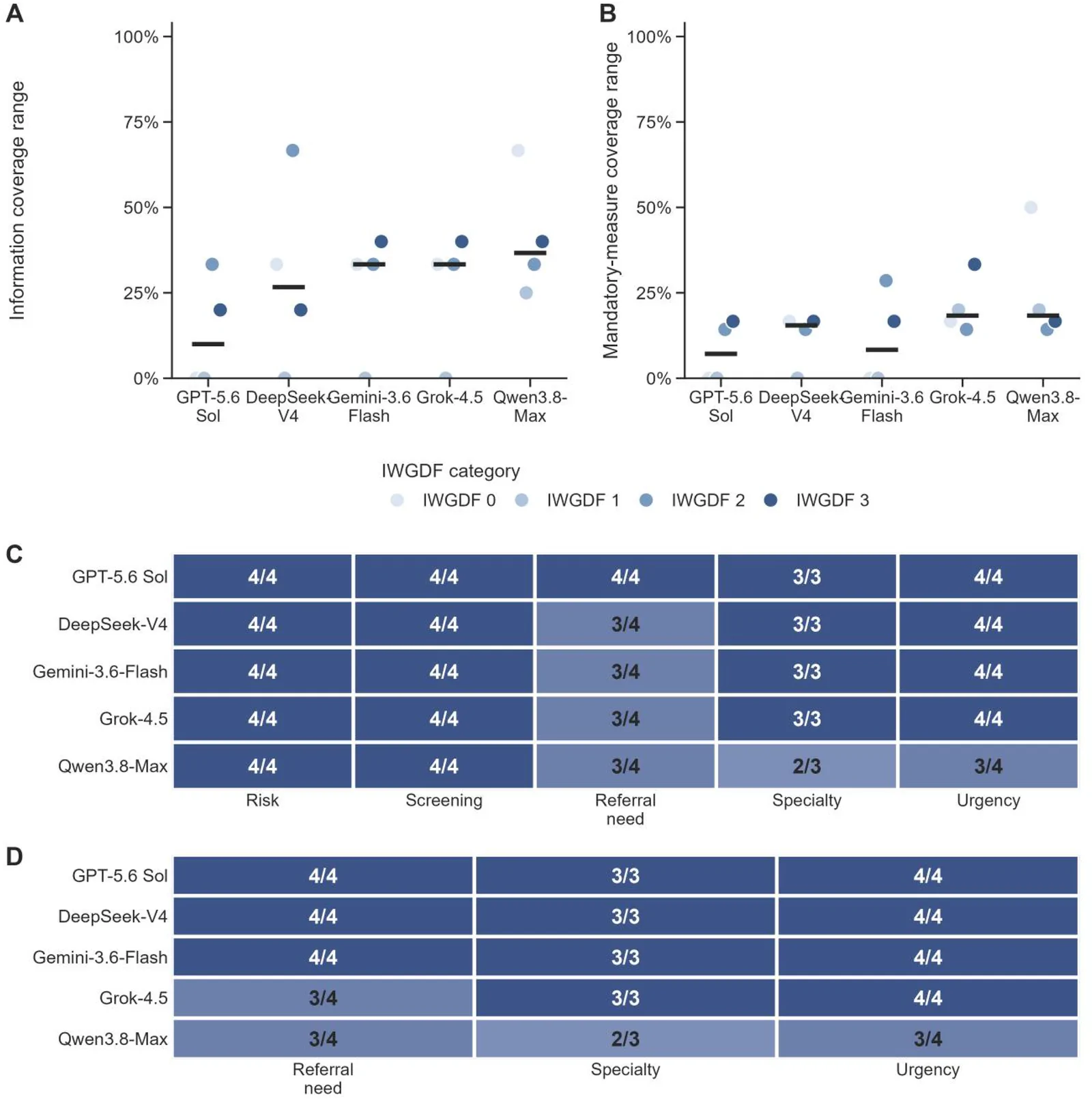
Exploratory repeated-generation concordance and variability across public LLM interfaces. **(A)**, within-combination range in information coverage, and **(B)**, within-combination range in mandatory-measure coverage across three independent generations. One case from each IWGDF category was repeated for each interface, yielding 20 interface–case combinations and 60 outputs. Coloured points indicate IWGDF categories, and black horizontal segments indicate interface medians. **(C)**, all-three-generation endpoint concordance, defined as all three generations matching the frozen reference standard. For referral outcomes, this is termed all-three-generation consensus concordance. **(D)**, binary consensus-concordance state consistency, defined as an unchanged concordant/discordant state across all three generations. Referral-specialty summaries included only eligible combinations. These findings are exploratory and hypothesis-generating; they should not be used for interface comparison or estimation of rare-failure rates. A stable discordant state is reproducible but not concordant with the frozen consensus standard. IWGDF, International Working Group on the Diabetic Foot; LLM, large language model.

Perfect agreement for risk category and screening frequency is clinically plausible due to the 2023 IWGDF system’s predominantly rule-based framework, especially once LOPS, PAD, foot deformity, previous ulcer or amputation, and end-stage renal disease have been identified (3). The benchmark was designed specifically to test this mapping: the underlying clinical findings were retained while explicit summary labels were removed, cases were consistently sequenced, and the prompt specified the IWGDF 2023 framework. Consequently, the findings indicate that under controlled conditions, contemporary public interfaces can effectively extract relevant features from curated text and apply the defined classification scheme. However, these findings do not imply that the models can predict incident ulceration, infer unmeasured findings, or independently derive a risk model. Furthermore, despite achieving 20 out of 20 correct classifications, the Wilson 95% CI included a lower limit of 83.9%, suggesting that ceiling performance in this pilot sample should not be interpreted as error-free or generalizable accuracy. Special caution is warranted for PAD assessment, as ankle-brachial index values may be falsely reassuring or noninterpretable in individuals with diabetes (9, 15). Cases with unresolved category-defining PAD uncertainty were excluded. The findings do not establish performance when vascular evidence is incomplete, internally discordant, or influenced by arterial incompressibility.

Referral performance exhibited notable heterogeneity and clinical relevance, with the discordance reflecting different clinical directions. For instance, in the case of LLM-009, three out of five interfaces recommended routine specialist referral, despite the frozen standard categorizing specialist referral as optional if the callus persisted or worsened. This scenario indicates a pattern of over-referral, raising concerns about resource utilization rather than risking missed necessary care. In contrast, for LLM-013, four out of five interfaces did not recommend the prespecified routine podiatry or specialist education referral, representing a case of under-referral. Because the frozen reference target was routine rather than urgent, these discordances should not be interpreted as failures to recognize an acute limb-threatening condition. Under the prespecified curated no-search benchmark conditions, no prespecified major safety errors were observed among the archived outputs; this finding does not establish clinical or autonomous safety. Notably, there was no referral discordance among category 2 outputs (**Figure 3**; **Supplementary Table S4**). The referral domain also posed challenges for the expert panel, which struggled to establish a consistent reference standard. Before reaching a consensus, all five experts agreed on the four-level referral disposition in only 11 out of 20 cases (55%). Additionally, five cases (25%) featured a 3:2 split in agreement, leading to an initial Fleiss kappa of approximately 0.57. In contrast, unanimous agreement was achieved for risk category and screening frequency across all cases. Referral decisions are complex and depend not only on IWGDF category but also on preulcerative findings, vascular uncertainties, patient self-care capacity, local service configurations, and the threshold for necessitating further assessment (16, 17). Referral agreement with the frozen consensus is an operational concordance measure, not proof of clinical correctness. Only one of the five 3:2 expert-vote cases showed any LLM referral discordance (LLM-009, 3/5 interfaces), whereas the highest LLM discordance occurred in a case with unanimous initial expert agreement (LLM-013, 4/5 interfaces). Greater expert disagreement therefore did not correspond to greater model discordance in this small descriptive sample; the sample is too small for formal association testing. The direction of referral discordance matters. Over-referral can consume specialist capacity and increase waiting times and cost; under-referral can delay vascular, neurological, or preventive assessment. Future evaluations should therefore report over-referral and under-referral separately rather than relying only on aggregate consensus agreement.

The completeness analyses revealed a distinct measurement challenge. Across the primary outputs, the median information-item coverage was 100% (IQR, 72.9%–100%), and the median mandatory-measure coverage was also 100% (IQR, 100%–100%). The information domain was designed to be broader than mere missing-data detection; in its frozen scoring units, it also encompassed explicit recognition that the available information was adequate for the current classification and for specific additional assessments or monitoring needs. Each scoring unit was treated as an indivisible rubric element, even when closely related clinical components were grouped together. However, prior to adjudication, the exact reviewer agreement for information coverage was only 60%, and for mandatory-measure coverage, it was 71%. The corresponding absolute-agreement, single-measure intraclass correlation coefficients were 0.661 and 0.344, respectively, with the CI for mandatory-measure coverage including 0. The median and IQR describe the distribution of completeness scores, whereas the ICC quantifies inter-reviewer reliability; both are necessary for interpretation. The high medians are descriptive, and the limited reliability indicates that apparent differences may reflect judgment in item mapping and denominator construction as well as model behavior. Completeness was therefore not used for interface ranking. Instead, they highlight the need for evaluations of open-ended recommendations to include prespecified scoring units, accepted semantic equivalents, multiple clinical reviewers, domain-specific reliability estimates, and transparent adjudication processes (14).

The exploratory, hypothesis-generating repeated-generation substudy further distinguished one-shot concordance from reproducibility. Across 20 interface-case combinations, all three generations were concordant with the frozen reference standard for risk category in 20/20 combinations and screening frequency in 20/20 combinations. All-three-generation consensus concordance was observed in 16/20 combinations for referral need, 14/15 eligible combinations for referral specialty, and 19/20 combinations for referral urgency. Binary consensus-concordance state consistency was observed in 18/20, 14/15, and 19/20 combinations, respectively. A combination could therefore be reproducible while remaining discordant with the frozen consensus standard. The median within-combination range was 33.3 percentage points for information coverage and 16.7 percentage points for mandatory-measure coverage. These sparse descriptive findings demonstrate that within-condition variation can occur, but they should not be used to compare interfaces or estimate failure probabilities, rare-event rates, or tail behavior (9, 18).

The absence of harmful recommendations or major safety errors should be interpreted only as the absence of a prespecified major safety phenotype under the recorded benchmark conditions. It does not establish autonomous safety or rule out rare harm. Active ulcers and other acute limb-threatening presentations were excluded; inputs were translated, curated, and consistently structured; the prompt prohibited fabrication and required explicit safety considerations; and no investigator-activated model-accessible internet search was used. These features reduced opportunities for dangerous failure and may not hold in routine clinical practice (9, 19).

### Comparison with prior work

The observed performance gradient aligns with a broader trend in clinical LLM evaluation: performance tends to be strongest when tasks can be reduced to explicit rule application and weaker when outputs require contextual judgment, prioritization, or locally contingent actions. In diabetes education, both base and retrieval-augmented models have been evaluated on common patient questions, emphasizing accuracy, comprehensiveness, and understandability (11). In treatment planning, GPT-4 has been compared with physicians using 50 anonymized patient records, uncovering residual concerns about plan completeness and safety (12). In diabetes-related foot disease, four models have been assessed against IWGDF/IDSA guidance for active infections (13). The present study extends these designs to prevention in the absence of active ulceration by employing paired real-world cases, a prospectively frozen multidisciplinary reference standard, and separate endpoints for classification, screening, referral, completeness, reproducibility, and safety.

The results introduce two qualifications to reports of high average LLM accuracy. First, aggregate performance can obscure case-specific clusters of errors: most referral discordance stemmed from a small subset of cases, including those where the experts themselves initially disagreed. Second, one-shot accuracy can mask instability across generations. The repeated-generation findings indicate that seemingly deterministic outputs may exhibit varying stability profiles across tasks, even within the same interface (20). Along with the limited reliability of completeness scoring, these observations challenge the validity of a single composite score or simple ranking of interfaces (21, 22). A clinically meaningful benchmark should report task-specific performance, case-level error concentration, generation-level stability, uncertainty in the human reference standard, and the potential consequences of each disagreement (23).

### Clinical implications

The findings advocate for a constrained, clinician-supervised role rather than autonomous decision-making. A clinical system could transform a completed foot examination into a draft IWGDF category, screening interval, checklist of information gaps and additional assessment needs, and a set of patient-specific prevention messages. Category-defining features and screening intervals are sufficiently rule-based to allow deterministic verification, while the LLM facilitates flexible synthesis and communication. The interface should provide an auditable draft rather than an authoritative final answer, displaying the case findings that support each classification and referral decision, flagging missing or contradictory data, allowing clinicians to revise recommendations, and retaining both the generated proposal and the clinician-approved actions in an audit trail. Clinician confirmation should remain mandatory for PAD interpretation, preulcerative lesions, potential Charcot features, contradictory or missing findings, and all referral decisions. Unconfirmed recommendations should not enter the clinical record or be communicated to patients (24, 25). Referral pathways should be configured locally, as acceptable specialties and urgency thresholds depend partly on service organization. This hybrid architecture reflects the observed task hierarchy: tasks closer to explicit guideline logic may be more suitable for constrained automation, while those requiring contextual judgment and resource-sensitive actions highlight the necessity for human oversight (25, 26).

### Strengths

This study presents several strengths. The patient, rather than the interface response, served as the independent clinical unit, and all interface comparisons were paired, as each evaluated the same cases. The case set, multidisciplinary reference standard, standardized prompt, scoring rules, repeated-generation plan, and statistical analysis were finalized prior to formal response collection. The first complete response was retained without follow-up prompting, selective regeneration, or best-of-response selection. Reviewers remained blinded to interface identity, and disagreements were adjudicated against case-specific scoring keys established before outputs were accessible. Classification, referral, completeness, reproducibility, and safety were reported as distinct domains rather than combined into an arbitrary composite score. Furthermore, the analysis avoided noninformative superiority testing when all interfaces achieved the same primary result and quantified uncertainty in both human scoring and repeated model generation. These design features mitigate selective reporting and clarify which conclusions are directly supported by the benchmark.

### Limitations

However, several limitations exist. First, this was a single-center exploratory benchmark involving 20 adults, with five cases deliberately sampled from each IWGDF risk stratum and no formal power calculation. Although this design ensured balanced representation across the risk spectrum, it provided limited precision: even with 20/20 observed concordance for each interface, the Wilson 95% CI had a lower bound of 83.9%, and one discordant classification would change an interface-level estimate by 5 percentage points and a stratum-specific estimate by 20 percentage points. Balanced sampling also altered the case mixture relative to the eligible pool, underrepresenting Category 0 and overrepresenting Category 3. Because 11/12 discordant referral outputs occurred in Categories 0 and 1, aggregate referral agreement is composition-dependent and may be more favorable than performance in a prevalence-representative cohort. We did not add a *post hoc* prevalence-weighted estimate because such an analysis was not prespecified and would apply population weights to unstable five-case stratum estimates. Accordingly, these findings do not establish near-perfect population performance or support precise stratum-specific estimates; larger, prevalence-representative, multicenter studies with prespecified weighting are required for generalizable inference.

Second, the cases were translated into English, anonymized, curated, and reformatted into a fixed clinical sequence, and records with unresolved category-defining neurological or vascular information were excluded. Although these choices supported paired comparison, they created idealized, best-case inputs by removing much of the chronology, redundancy, missingness, and internal inconsistency of routine records. The benchmark may therefore overestimate performance with fragmented, redundant, multilingual, or internally inconsistent clinical documentation and does not establish performance when vascular evidence is incomplete, internally discordant, or influenced by arterial incompressibility. External validation should use prospectively collected raw records, prespecified translation procedures, and language-specific evaluations.

Third, cases involving active ulceration and acute limb-threatening presentations were excluded, and the models did not receive images, raw vascular studies, or longitudinal data. While these exclusions helped avoid inappropriate extrapolation from a prevention benchmark, they confine the findings to ulcer-risk stratification and prevention among clinically stable cases.

Fourth, the evaluated systems were timestamped public web interfaces rather than stable, fully auditable model snapshots. Provider-side routing, hidden system instructions, unexposed retrieval, memory, personalization, and subsequent updates could not be fully controlled. The study minimized this source of variation by recording the interface and access conditions, using an identical frozen prompt, retaining the first complete response, and conducting a prespecified repeated-generation substudy. Nevertheless, the results characterize the accessed interface versions under those conditions, rather than a fixed underlying model. The no-search condition limits external validity for workflows in which an LLM retrieves current guidelines or institution-specific pathways. The present results characterize a controlled baseline condition and should not be generalized to retrieval-enabled systems. This reliance on public interfaces highlights the necessity for versioned, auditable model snapshots, standardized change logs, and revalidation procedures for clinical evaluation; without these, reliable attribution of performance changes to the model, interface, provider-side routing, or local implementation remains challenging. Any material changes in these components should prompt revalidation.

Fifth, the repeated-generation substudy was restricted to one prespecified case per IWGDF category, three generations, and 20 interface-case combinations. Its consensus-concordance counts demonstrate that instability can occur, but are too sparse to provide precise stability estimates, estimate rare failures, compare interfaces, or characterize tail behavior.

Sixth, referral standards relied on expert consensus, and the limited reliability of mandatory-measure coverage diminished the precision of open-ended scoring. The prospective freezing of the reference standard and scoring key prevented adaptations to model outputs, while duplicate reviews and adjudications reduced individual rater effects. Nonetheless, the reviewers did not utilize a separate calibration set, and neither adjudication nor a scoring manual eliminated construct uncertainty. Additionally, while interface metadata were removed, the original wording, structure, length, and citations were retained; thus, stylistic cues or self-identifying language may have allowed inferences about interface identity, making blinding an attempted rather than guaranteed measure. Future benchmarks should implement calibrated scoring, assess blinding success, retain expert disagreement information, and differentiate clinically unsafe decisions from reasonable alternatives.

Lastly, this benchmark measured agreement with a frozen reference standard rather than prospective ulcer incidence, clinician performance, workflow efficiency, patient understanding, equity, or clinical outcomes. The repeated outputs were clustered within selected cases, and neither clinicians nor patients received the generated recommendations. Consequently, the study cannot establish clinical effectiveness, demonstrate noninferiority to multidisciplinary care, or confirm readiness for independent deployment. This limitation is inherent to the noninterventional design and can only be addressed through prospective clinical evaluation.

### Future directions

Future external validation should evaluate these systems prospectively across multiple centers and real clinical workflows using substantially larger, prevalence-representative cohorts and prespecified prevalence-weighted analyses, with sufficient cases per stratum to estimate referral performance without the 20-percentage-point granularity of the present pilot. Test sets should encompass incomplete and internally discordant examinations, raw clinical documentation, multiple languages, multimodal inputs, evolving preulcerative findings, and transitions from prevention to active diabetic foot disease. Repeated-generation designs should be expanded to report worst-case as well as average performance, with referral errors categorized into over-referral and under-referral and weighted according to likely clinical consequences. Reference standards should preserve expert disagreement rather than forcing every case into an artificially certain label. Future studies should preserve initial expert vote distributions, report agreement with the initial majority vote as an auxiliary metric, and prespecify over-referral and under-referral as distinct outcomes. Controlled implementation studies should assess whether clinician-LLM collaboration enhances documentation completeness, appropriate referral, time to specialist assessment, and patient education without increasing workload, inequity, unnecessary referrals, or alert fatigue. Clinical outcome studies are required to determine whether any process improvements lead to reduced ulcerations or amputations. External validation, deterministic checks for rule-based outputs, local pathway configuration, and prespecified safety monitoring should precede any increase in system autonomy. Future studies should compare the recorded no-search condition with controlled retrieval-augmented generation using a fixed, versioned guideline corpus and local clinical pathways, prespecified retrieval settings, and auditable source provenance. Such studies should assess whether retrieval improves currency and local applicability without introducing unsupported source selection, citation errors, or additional variability.

### Conclusions

In this paired pilot benchmark, five public LLM interfaces accurately reproduced IWGDF risk categories and screening intervals for all 20 curated cases, but referral agreement and management reproducibility were lower. The findings support clinician-supervised, auditable decision support, but do not establish clinical effectiveness or autonomous safety.

## Data Availability

The original contributions presented in the study are included in the article/**Supplementary Material**. Further inquiries can be directed to the corresponding authors.
